# Age-Related Patterns of Myocardial Recovery After Primary PCI for Acute Myocardial Infarction: A Prospective 12-Month Study

**DOI:** 10.3390/biomedicines14091895

**Published:** 2026-08-25

**Authors:** Bogdan-Sorin Tudurachi, Larisa Anghel, Andreea Tudurachi, Mircea Ovanez Balasanian, Radu Andy Sascău, Cristian Stătescu

**Affiliations:** 1 Internal Medicine Department, “Grigore T. Popa” University of Medicine and Pharmacy, 700503 Iași, Romania; bogdan-sorin.tudurachi@d.umfiasi.ro (B.-S.T.); leonte.andreea@d.umfiasi.ro (A.T.); ovanes718@yahoo.com (M.O.B.); radu.sascau@umfiasi.ro (R.A.S.); cristian.statescu@umfiasi.ro (C.S.); 2 Cardiology Department, Cardiovascular Diseases Institute “Prof. Dr. George I. M. Georgescu”, 700503 Iași, Romania

**Keywords:** acute myocardial infarction, primary percutaneous coronary intervention, aging, ventricular remodeling, global longitudinal strain, guideline-directed medical therapy

## Abstract

**Background/Objectives:** Age may influence myocardial recovery after acute myocardial infarction (AMI) beyond changes in left ventricular ejection fraction (LVEF). We investigated age-related patterns in ischemic delay, admission biomarkers, biventricular function, myocardial deformation, and exploratory patterns of prescribed heart failure guideline-directed medical therapy (HF-GDMT) classes after primary percutaneous coronary intervention (PCI). **Methods:** This prospective frequency-matched cohort study included 90 AMI patients treated with primary PCI, stratified into younger (25–44 years; *n* = 30) and older (≥45 years; *n* = 60) groups. Clinical data, ischemic time, cardiac biomarkers, serial echocardiography at baseline, 6, and 12 months, and HF-GDMT intensity were analyzed. **Results:** Older patients had longer pain-to-balloon time (720 [480–1230] vs. 540 [420–660] min; *p* = 0.021), higher hs-cTnI (*p* = 0.045), and higher NT-proBNP (4776 [3013–5952] vs. 833.5 [95.7–1939.5] pg/mL; *p* < 0.001). Baseline and 12-month LVEF were similar between groups. Younger patients showed greater 12-month global longitudinal strain (GLS) improvement (−3.7 [−5.1 to −1.9] vs. −2.3 [−3.0 to −1.3] percentage points; *p* = 0.002), despite comparable absolute GLS. In exploratory post hoc analyses, prescribed HF-GDMT drug-class count showed an association with LVEF change primarily in older patients, who also had significantly lower baseline LVEF (ρ = 0.59; *p* < 0.001). **Conclusions:** In this prospective exploratory cohort, 12-month conventional systolic recovery assessed by LVEF and absolute GLS was comparable between age groups after primary PCI. However, the older comparator group had longer ischemic delay and higher admission biomarkers reflecting cardiomyocyte injury and myocardial wall stress, while younger patients showed greater longitudinal GLS improvement over time. These findings suggest that age-stratified recovery after AMI is better characterized by ischemic timing, baseline biomarker profile, and deformation trajectory than by LVEF alone. Because infarct size was not directly quantified, admission biomarker differences should not be interpreted as evidence of larger infarcts.

## 1. Introduction

Acute myocardial infarction (AMI) remains a leading cause of morbidity and mortality worldwide, despite substantial advances in reperfusion strategies, particularly primary percutaneous coronary intervention (PCI). Age is an important determinant of both the clinical presentation and the subsequent remodeling phenotype following acute ischemic injury. From a clinical perspective, AMI in younger adults (25–44 years) is frequently characterized by single-vessel disease and specific metabolic risk profiles such as smoking and obesity, whereas older adults (≥45 years) within the premature coronary artery disease spectrum more commonly present with multi-vessel involvement, a higher comorbidity burden, and notably, prolonged pre-hospital delays. These age-related differences may contribute to heterogeneous patterns of myocardial injury, ventricular remodeling, and long-term prognosis.

Although timely restoration of coronary blood flow limits infarct expansion and improves survival, a considerable proportion of patients remain at risk of adverse left ventricular remodeling, heart failure (HF), and major adverse cardiovascular events (MACE). Therefore, early and accurate risk stratification is essential for identifying patients at increased risk and for optimizing post-infarction management [1,2,3]. Even in the context of standardized contemporary treatment, post-AMI recovery is strongly influenced by several key determinants, particularly the time from symptom onset to mechanical reperfusion. Pain-to-balloon time represents a clinically relevant marker of total ischemic duration and is closely related to the extent of myocardial necrosis, residual myocardial viability, and subsequent ventricular remodeling [4]. Prolonged delay in reperfusion may increase myocardial injury and ventricular wall stress, as reflected by elevated high-sensitivity cardiac troponin I (hs-cTnI) and N-terminal pro-B-type natriuretic peptide (NT-proBNP), biomarkers that respectively indicate cardiomyocyte necrosis and hemodynamic/neurohormonal stress. A comprehensive assessment of post-infarction remodeling requires integration of conventional echocardiographic indices with more sensitive parameters of myocardial performance. While LVEF remains a central measure of systolic function, GLS provides additional information regarding subclinical myocardial dysfunction and may detect impaired contractile performance before overt changes in ejection fraction become apparent. Similarly, tricuspid annular plane systolic excursion (TAPSE) offers a practical and clinically relevant assessment of right ventricular longitudinal function, which may influence global hemodynamic status and post-AMI recovery [5,6,7,8,9].

In accordance with epidemiological frameworks and consensus definitions from the World Health Organization and premature coronary artery disease registries, which conventionally define premature myocardial infarction as occurring under the age of 45–50, the present study utilized a strict 45-year threshold to distinguish true premature presentations from older comparator strata. The aim of this multi-modal, exploratory investigation was to evaluate the baseline clinical characteristics, pre-hospital ischemic intervals, acute biomarker footprints, and 12-month serial biventricular echocardiographic tracking in younger (25–44 years) versus older (≥45 years) cohorts undergoing primary PCI, while concurrently exploring baseline prescription patterns of guideline-directed medical therapy classes.

The novelty of this study lies in the integration of ischemic delay, admission biomarker profile, serial biventricular echocardiography, deformation imaging, and exploratory HF-GDMT prescription class counts in an age-stratified post-AMI cohort. This approach allows recovery after primary PCI to be characterized beyond LVEF alone and highlights the importance of trajectory-based assessment in younger versus older comparator patients.

## 2. Materials and Methods

### 2.1. Study Design and Population

**Figure 1:**
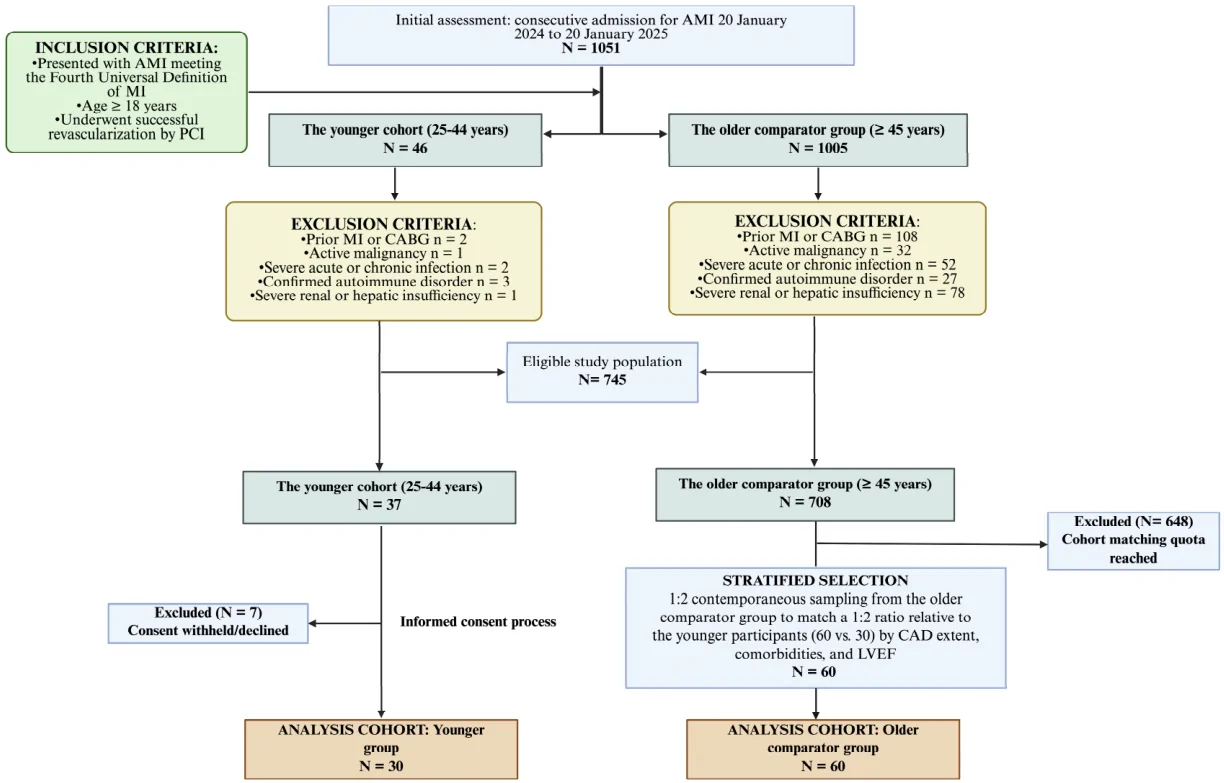
Study flow diagram illustrating patient screening, eligibility, and age-stratified cohort allocation. AMI, acute myocardial infarction; CABG, coronary artery by-pass grafting; CCS, chronic coronary syndrome; LVEF, left ventricular ejection fraction; MI, myocardial infarction; PCI, percutaneous coronary intervention.

This prospective, frequency-matched cohort study was conducted in the Surveillance and Advanced Treatment Unit for Critical Cardiac Patients of the “Prof. Dr. George I.M. Georgescu” Institute of Cardiovascular Diseases, Iași, Romania. Consecutive patients admitted with AMI between 20 January 2024 and 20 January 2025 were prospectively screened (*n* = 1051, comprising *n* = 46 patients aged 25–44 years and *n* = 1005 patients aged ≥45 years). To balance baseline anatomical and functional characteristics, older comparator patients (≥45 years, *n* = 60) were enrolled using a 1:2 frequency-matching approach based on coronary lesion distribution, cardiovascular comorbidity burden, and baseline LVEF (Figure 1).

Patients were eligible for inclusion if they met all of the following criteria: diagnosis of AMI according to the Fourth Universal Definition of Myocardial Infarction; age between 25 and 44 years for the young group or ≥45 years for the older comparator group; successful revascularization by PCI during the index hospitalization; and provision of written informed consent. Exclusion criteria were previous myocardial infarction, prior coronary artery bypass grafting, active malignancy, severe acute or chronic infection, autoimmune disease, and severe renal or hepatic insufficiency. These conditions were excluded because they may independently affect systemic inflammation, biomarker expression, ventricular function, and hematological inflammatory indices, which have recognized associations with myocardial necrosis and ischemic burden in acute settings [10], thereby potentially confounding the interpretation of post-AMI inflammatory and remodeling trajectories.

Currently, there is no standard definition for premature coronary heart disease (CHD), and varying cut-offs, ranging from 40 to 55 years, have been used in the previous literature [10]. In the present study, a strict cutoff of 45 years (25–44 years for the young cohort vs. ≥45 years for the older comparator group) was defined a priori based on two main considerations. First, it maps directly onto demographic schemes used by the World Health Organization and United Nations that define young adulthood as the period from 25 to 44 years. Secondly, the cut-off age of 44 years was to define a distinct clinical and pathophysiologic phenotype of early-onset myocardial infarction, without the confounding overlap of cumulative age-related multi-vessel atherosclerosis and vascular senescence usually seen after the fifth decade of life. Myocardial infarction frequently serves as the initial clinical manifestation irrespective of patient age; nevertheless, comprehensive epidemiological data concerning this specific younger demographic remain sparse. Consequently, documented incidence estimates demonstrate marked variance, heavily contingent upon the underlying source population, the observational timeframe, and the exact diagnostic criteria applied [11]. Following initial screening, 306 patients were excluded based on predefined exclusion criteria: 9 patients in the younger cohort (prior MI or CABG, *n* = 2; active malignancy, *n* = 1; severe acute or chronic infection, *n* = 2; confirmed autoimmune disorder, *n* = 3; severe renal or hepatic insufficiency, *n* = 1) and 297 patients in the older cohort (prior MI or CABG, *n* = 108; active malignancy, *n* = 32; severe acute or chronic infection, *n* = 52; confirmed autoimmune disorder, *n* = 27; severe renal or hepatic insufficiency, *n* = 78). Consequently, 745 patients met all inclusion criteria (*n* = 37 young patients and *n* = 708 older patients). In the younger group, 7 patients declined or withheld consent during the informed consent process, yielding a final analyzed young cohort of *n* = 30 (Figure 1). Patients were stratified according to age into two predefined groups: a young AMI group, comprising patients aged 25–44 years (*n* = 30), and an older comparator group, comprising patients aged ≥45 years (*n* = 60). All enrolled patients were prospectively followed up after the index AMI, with scheduled clinical, biochemical, therapeutic, and echocardiographic reassessments performed at 6 and 12 months. This longitudinal design allowed the evaluation of age-related differences in post-infarction recovery, biventricular functional dynamics, biomarker profiles, and the exploratory association between prescribed guideline-directed medical therapy classes and subsequent cardiac remodeling.

The older comparator group included eligible patients aged ≥45 years admitted during the same study period. To ensure balanced baseline anatomical and functional profiles while maintaining feasibility for intensive biomarker profiling and serial 12-month echocardiographic follow-up, a 1:2 frequency-matching approach was employed. From the 708 eligible patients aged ≥45 years, 60 comparator patients were recruited to match the young group on coronary lesion extent, cardiovascular comorbidity burden, and baseline LVEF. The remaining 648 eligible older patients were not included due to fulfillment of the predefined cohort quota. All enrolled patients originating from the same clinical setting underwent identical diagnostic and PCI-based management workflows and were followed under the same prospective protocol. Nonetheless, due to the observational nature of the study, residual confounding cannot be entirely ruled out, and all age-stratified functional comparisons are considered exploratory.

The diagnosis of myocardial infarction was established according to the Fourth Universal Definition of Myocardial Infarction. Patients were further classified as having ST-segment elevation myocardial infarction (STEMI) or non-ST-segment elevation myocardial infarction (NSTEMI) based on clinical presentation, electrocardiographic findings, and biomarker dynamics. New-onset left bundle branch block fulfilling Sgarbossa criteria was classified as STEMI, in accordance with accepted diagnostic recommendations [12,13].

The study protocol was reviewed and approved by the Ethics Committee of “Grigore T. Popa” University of Medicine and Pharmacy of Iași on 5 December 2023, approval number 365/2023. The study was conducted in accordance with the principles of the Declaration of Helsinki. All participants provided written informed consent prior to enrollment and before any study-specific data collection.

### 2.2. Clinical and Paraclinical Data

Baseline demographic data and cardiovascular risk factors were collected from medical records and structured clinical assessment. Variables included age, sex, hypertension, obesity, body mass index, known dyslipidemia, diabetes mellitus, smoking status, alcohol consumption, drug use and AMI type. The STEMI presentation was recorded as a categorical variable. For patients presenting with STEMI, revascularization was performed via emergent primary PCI. For the subset of patients presenting with NSTEMI (16.7% of the overall cohort), inclusion was strictly restricted to individuals classified as ‘very high-risk’ according to current ESC guidelines, exhibiting features such as hemodynamic instability, electrical failure, or refractory ischemic chest pain. Consequently, these very high-risk NSTEMI patients underwent an immediate invasive strategy within less than 2 h of admission, using the exact same operational 24/7 emergency catheterization workflow as the STEMI cohort. To maintain methodological consistency across the entire sample, the total ischemic duration—operationalized as pain-to-balloon time—was calculated identically for both STEMI and very high-risk NSTEMI participants as the total time elapsed between the patient’s earliest clinical manifestations of acute ischemia and the mechanical restoration of coronary flow via the first catheter balloon expansion. The Cardiovascular Diseases Institute in Iași is a high-volume tertiary referral center equipped with a dedicated, 24/7 primary PCI program and a permanent active-duty team for interventional cardiology. Consequently, in-hospital processing is highly optimized and standardized, with all included AMI patients undergoing mechanical reperfusion within the first hour of hospital admission (door-to-balloon time < 60 min), regardless of age.

Blood samples were obtained at admission or during the index hospitalization according to institutional protocols. All patients underwent primary PCI according to standard interventional practice.

Post-AMI pharmacological therapy was documented at discharge and confirmed during scheduled follow-up visits. To characterize the breadth of medical therapy, a pragmatic HF-GDMT class count (ranging from 0 to 4) was recorded based on the concurrent prescription of the four foundational prognostic classes: (1) renin–angiotensin system inhibitors (ACEi/ARB/ARNI), (2) evidence-based beta-blockers, (3) mineralocorticoid receptor antagonists (MRAs), and (4) sodium–glucose cotransporter-2 (SGLT2) inhibitors. Importantly, this score represents a pragmatic count of prescribed/initiated drug classes rather than a direct verification of true patient adherence (e.g., pharmacy-refill metrics), individual drug tolerability, or longitudinal titration toward guideline-recommended target doses.

Transthoracic echocardiography was performed at baseline, 6 months, and 12 months using standard two-dimensional, Doppler, and tissue Doppler techniques, in accordance with current ASE/EACVI recommendations. All examinations were performed using a General Electric Healthcare Vivid E95 system, and offline analyses were conducted with EchoPAC software (version 13.1.1). Left ventricular ejection fraction was calculated using the biplane Simpson method from apical four- and two-chamber views whenever image quality permitted. Global longitudinal strain was derived from standard apical acquisitions by two-dimensional speckle-tracking echocardiography and expressed as a negative percentage, with more negative values indicating better longitudinal myocardial deformation. Right ventricular longitudinal function was assessed by tricuspid annular plane systolic excursion measured in the apical four-chamber view using M-mode alignment with the lateral tricuspid annulus. The mean E/E′ ratio was used as a surrogate marker of left ventricular filling pressure, while left ventricular outflow tract velocity–time integral was considered an index of forward stroke distance. To ensure maximum consistency, all comprehensive echocardiographic examinations and offline text analyses were completed by a single investigator who was fully blinded to clinical parameters and follow-up intervals. The intra-observer variability for conventional and deformation indices was strictly validated to meet international quality controls. The investigator’s measurement variability remained thoroughly below the maximum thresholds permitted by the ASE/EACVI guidelines (specifically, <7% for biplane Simpson LVEF and <1.5 percentage points for structural GLS assessment), confirming optimal test–retest reliability across the prospective study framework. The main echocardiographic variables analyzed were LVEF, GLS, TAPSE, mean E/E′ ratio, and LVOT VTI. The principal outcomes were age-related differences in baseline clinical and biochemical profiles, together with serial echocardiographic recovery after acute myocardial infarction. Left ventricular recovery was assessed using follow-up LVEF and GLS values and their absolute change from baseline to 12 months when available. Right ventricular and diastolic recovery were evaluated through TAPSE and mean E/E′ ratio trajectories, respectively. The relationship between prescribed guideline-directed medical therapy class count and 12-month LVEF trajectory was explored as an exploratory, hypothesis-generating analysis.

### 2.3. Statistical Analysis

Statistical analyses were performed using IBM SPSS Statistics, version 26.0 (IBM Corp., Armonk, NY, USA). The distribution of continuous variables was assessed using the Kolmogorov–Smirnov test and by visual inspection of histograms and Q–Q plots. Continuous variables were reported as mean ± standard deviation when normally distributed and as median with interquartile range [IQR] when non-normally distributed. Categorical variables were expressed as absolute frequencies and percentages.

Patients were stratified into two predefined age groups: young patients aged 25–44 years and older patients aged ≥45 years. Between-group comparisons were performed using the independent-samples *t* test for normally distributed continuous variables and the Mann–Whitney U test for non-normally distributed variables. Categorical variables, including sex, cardiovascular risk factors, STEMI presentation, and treatment-related variables, were compared using the chi-square test or Fisher’s exact test, as appropriate.

Baseline clinical characteristics, cardiovascular risk factors, ischemic time, cardiac biomarkers, and echocardiographic parameters were compared between the two age groups. The analyzed biomarkers included high-sensitivity cardiac troponin I and N-terminal pro-B-type natriuretic peptide. Echocardiographic parameters included left ventricular ejection fraction, global longitudinal strain, tricuspid annular plane systolic excursion, mean E/E′ ratio, and left ventricular outflow tract velocity–time integral. These variables were assessed at baseline, 6 months, and 12 months, according to the prospective follow-up protocol.

Serial echocardiographic changes were evaluated by comparing values obtained at baseline, 6 months, and 12 months within each age group. For non-normally distributed repeated measurements, the Friedman test was used, followed by post hoc pairwise comparisons with the Wilcoxon signed-rank test when appropriate. Between-group differences at each follow-up time point were assessed using the Mann–Whitney U test. Changes in functional parameters over time were additionally expressed as absolute differences between follow-up and baseline values, including LVEF recovery and GLS improvement at 12 months.

The exploratory analysis of prescribed HF-GDMT drug classes and LVEF changes was assessed by comparing LVEF changes according to the number of HF drug classes administered during follow-up. For this analysis, LVEF recovery was defined as the absolute difference between LVEF at 12 months and baseline LVEF. Depending on data distribution and group size, comparisons across GDMT intensity categories were performed using the Kruskal–Wallis test or Mann–Whitney U test. Correlations between GDMT intensity and LVEF recovery were explored using Spearman’s rank correlation coefficient. The exploratory association between post-infarction medical therapy and left ventricular functional changes was evaluated using the pragmatic HF-GDMT prescription score (0 to 4 classes). For this exploratory analysis, LVEF recovery was defined as the absolute change between 12-month and baseline LVEF (ΔLVEF). Differences across prescription strata (lower-intensity [0–2 classes] vs. higher-intensity [3–4 classes]) were tested using the Mann–Whitney U test, and correlations were assessed using Spearman’s rank correlation coefficient. Given that prescription counts reflect baseline clinical indication and functional severity rather than verified patient adherence, drug tolerability, or target dose optimization, all treatment-related analyses are presented strictly as exploratory and hypothesis-generating.

Effect sizes were calculated for the principal between-group comparisons. For continuous variables, Mann–Whitney r was used, while Cramér’s V was used for categorical variables. The main between-group effects were small to moderate for pain-to-balloon time (r = 0.24), hs-cTnI (r = 0.21), baseline TAPSE (r = 0.22), and baseline E/E′ (r = 0.22), and large for NT-proBNP (r = 0.65). The effect size for ΔGLS was moderate at both 6 months (r = 0.38) and 12 months (r = 0.33). All core echocardiographic parameters were analyzable in the full cohort at each time point, with 100% GLS feasibility.

Because of the small sample size (*n* = 90) and the relatively small number of patients in the young AMI group (*n* = 30), a multivariable regression model was not performed, as proper adjustment for potential confounders was not possible without the risk of overfitting the model. Thus, all analyzes are to be considered exploratory and hypothesis-generating. Because a frequency-matching design (group-level matching) was used to align baseline clinical and angiographic features rather than individual pair-matching, between-group comparisons for continuous variables were appropriately performed using the independent-samples *t*-test or the Mann–Whitney U test, and categorical variables were compared using the chi-square or Fisher’s exact test. A two-sided *p* value < 0.05 was considered statistically significant.

## 3. Results

### 3.1. Baseline Characteristics and Cardiac Biomarkers

The study cohort included 90 patients with acute myocardial infarction treated with primary percutaneous coronary intervention, comprising 30 younger patients and 60 older patients. The proportion of female patients was higher in the older cohort, although this difference did not reach statistical significance (28.3% vs. 13.3%, *p* = 0.113).

The distribution of conventional cardiovascular risk factors was largely comparable between the two age groups. Hypertension was documented in 33.3% of younger patients and 43.3% of older patients (*p* = 0.361), while obesity was present in 23.3% and 28.3% of patients, respectively (*p* = 0.613). The prevalence of diabetes mellitus was identical in both groups, affecting 13.3% of patients. Smoking was highly prevalent across the entire cohort, with similar rates in younger and older patients (76.7% vs. 78.3%, *p* = 0.858). Likewise, the proportion of patients presenting with ST-segment elevation myocardial infarction was identical between groups, being observed in 83.3% of patients in each age category (*p* = 1.000). Baseline angiographic characteristics were comparable between the two study groups. No statistically significant differences were observed with respect to infarct location (*p* = 0.357), culprit vessel distribution (*p* = 1.000), or the extent of coronary artery disease, expressed as single-, two-, or three-vessel involvement (*p* = 1.000). Anterior myocardial infarction represented the predominant infarct territory in both groups, while the distribution of culprit coronary arteries and angiographic disease burden remained balanced between younger and older patients.

Despite broadly similar baseline risk-factor profiles and comparable AMI presentation patterns, older patients experienced a significantly longer pain-to-balloon time compared with younger patients (720 [480–1230] min vs. 540 [420–660] min, *p* = 0.021). When evaluating the cohort based on the clinical guideline window, the proportion of individuals exhibiting a late presentation (>12 h from symptom onset to balloon) was significantly higher in the older group compared to the younger cohort (45.0% [27/60] vs. 20.0% [6/30], *p* = 0.022), as shown in Table 1. 

This prolonged ischemic interval was paralleled by significantly higher circulating markers of myocardial injury and ventricular wall stress. High-sensitivity cardiac troponin I levels were higher in the older group than in the younger group (2757.5 [2044.0–4045.3] vs. 914.5 [171.5–4213.5], *p* = 0.045), while NT-proBNP concentrations were markedly elevated among older patients (4776.0 [3013.3–5952.0] pg/mL vs. 833.5 [95.7–1939.5] pg/mL, *p* < 0.001) (Table 2). 

Collectively, these findings indicate that older patients presented with a more unfavorable acute biomarker profile alongside longer ischemic exposure, despite a similar frequency of STEMI presentation. However, given the observational, age-stratified design and the lack of serial peak/cumulative biomarker measurements or cardiac magnetic resonance imaging, these marked admission differences—particularly in NT-proBNP—cannot be assumed to reflect greater infarct-related myocardial stress alone, or direct evidence of larger final infarct size, as pre-existing age-dependent neurohormonal variations and subclinical structural changes may also contribute. Circulating admission levels are heavily influenced by physiological aging, baseline ventricular compliance, subclinical diastolic alterations, and altered renal clearance. Furthermore, in the absence of serial peak/cumulative troponin kinetics or cardiac magnetic resonance (CMR) imaging, single admission biomarker measurements cannot establish or quantify differences in final infarct size.

### 3.2. Echocardiographic Remodeling and Functional Recovery After AMI

At baseline, conventional left ventricular systolic function was similarly impaired in both groups. Median LVEF was 45% in younger patients and 45% in older patients, with no significant between-group difference. Baseline GLS was also comparable between younger and older patients, with values of −11.8 [−13.1 to −10.5]% and −11.2 [−13.2 to −9.2]%, respectively (*p* = 0.144), indicating a similar initial degree of impairment in longitudinal left ventricular systolic deformation. In contrast, statistically significant but clinically modest between-group differences were observed in TAPSE and mean E/E′ ratio at baseline. Both parameters remained within generally accepted reference ranges, indicating that these differences should be interpreted as subclinical variations rather than overt right ventricular dysfunction or elevated filling pressures. TAPSE was significantly lower in older patients compared with younger patients (20 [17–22] mm vs. 21.5 [19–23] mm, *p* = 0.034), while the mean E/E′ ratio was significantly higher in the older group (8.4 [7.3–11.2] vs. 7.5 [6.5–10.1], *p* = 0.042). LVOT VTI was numerically lower in older patients, although this difference did not reach statistical significance (18.6 [16.1–21.1] cm vs. 20.0 [17.3–22.4] cm, *p* = 0.063). At 6 months, both groups demonstrated improvement in conventional left ventricular systolic function, with median LVEF increasing to 50% in younger patients and 50% in older patients (*p* = 0.473). GLS also improved in both groups, reaching −16.0 [−17.6 to −13.8]% in younger patients and −14.5 [−16.4 to −12.5]% in older patients, without a statistically significant difference in absolute GLS values at this time point (*p* = 0.114). However, right ventricular recovery appeared to be slower in the older cohort, as TAPSE remained significantly lower in older patients at 6 months (20.5 [18–22.2] mm vs. 22 [20.2–23] mm, *p* = 0.012). Conversely, the baseline difference in estimated filling pressure was no longer evident, with comparable mean E/E′ ratios in younger and older patients (6.9 [6.4–8.1] vs. 7.2 [5.9–8.7], *p* = 0.969). LVOT VTI also remained similar between groups (*p* = 0.264).

**Figure 2:**
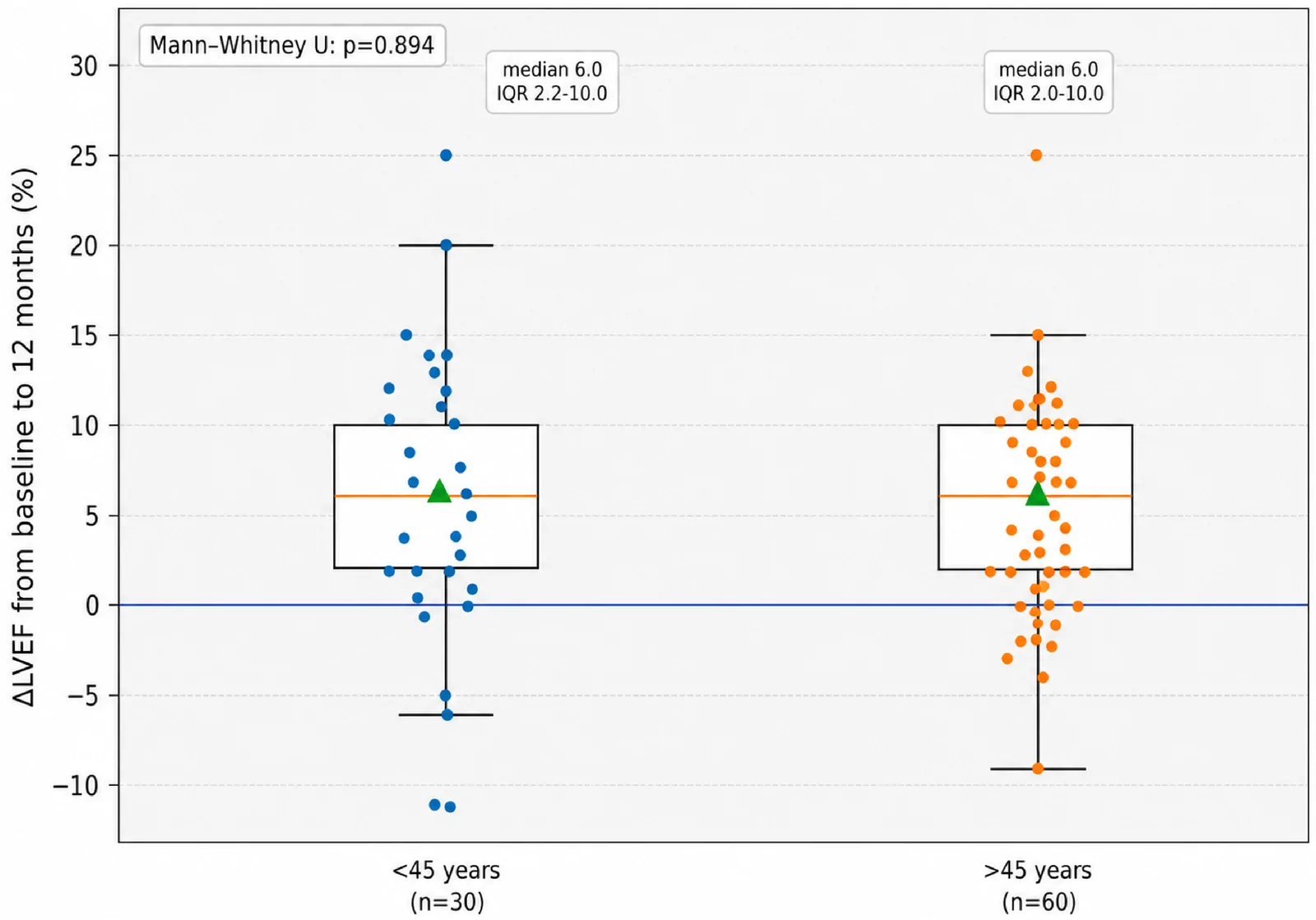
Age-stratified left ventricular ejection fraction recovery at 12 months after acute myocardial infarction. Box plots illustrate the distribution of absolute change in left ventricular ejection fraction (LVEF, percentage points) from baseline to 12 months in younger (<45 years, *n* = 30) versus older (≥45 years, *n* = 60) patients. Horizontal orange lines indicate median values, green triangles represent group means, box boundaries define the interquartile range (IQR, 25th–75th percentiles), and whiskers extend to the minimum and maximum observed values. Individual data points represent single patients. Between-group comparison was evaluated using the Mann–Whitney U test (*p* = 0.894). ΔLVEF, change in LVEF from baseline.

At 12 months, median LVEF was similar in both age groups (younger: 50 [45–55]%; older: 50 [45–55]%; *p* = 0.777), indicating comparable recovery of conventional left ventricular systolic function (Figure 2). At baseline, the overall cohort (*n* = 90) included 31 patients (34.4%) with HFrEF (LVEF ≤ 40%), 32 (35.6%) with HFmrEF (LVEF 41–49%), and 27 (30.0%) with HFpEF (LVEF ≥ 50%). The distribution of LVEF categories was comparable between the age groups, with 9 HFrEF, 12 HFmrEF, and 9 HFpEF patients in the younger cohort and 22 HFrEF, 20 HFmrEF, and 18 HFpEF patients in the older cohort.

Among patients fulfilling the baseline HFrEF definition (*n* = 31), HFimpEF was defined according to the 2021 Universal Definition of Heart Failure as baseline LVEF ≤ 40%, an absolute increase in LVEF of ≥10 percentage points, and a follow-up LVEF > 40%. Within this subgroup, 6 of 9 younger patients (66.7%) and 7 of 22 older patients (31.8%) met the criteria for HFimpEF, whereas 3 of 9 (33.3%) and 15 of 22 (68.2%), respectively, remained in the HFrEF category at 12 months. Given the limited number of patients meeting baseline HFrEF criteria, these findings should be considered exploratory and descriptive. Most patients in this subgroup (29/31) received high-intensity GDMT (3–4 drug classes) during follow-up, reflecting standard indication-driven guideline implementation for reduced ejection fraction.

**Figure 3:**
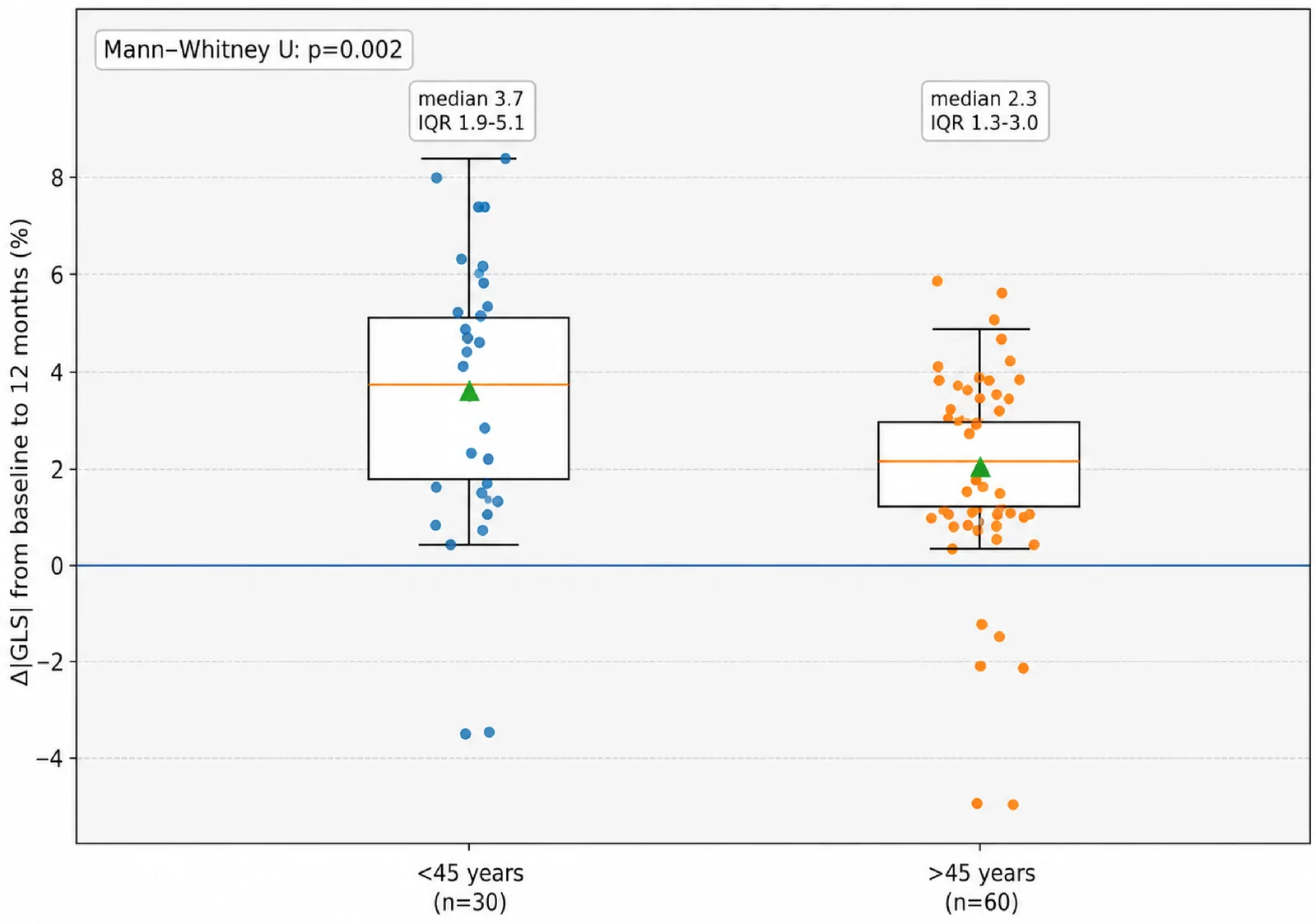
Age-stratified global longitudinal strain recovery at 12 months after acute myocardial infarction. Box plots show the absolute change in global longitudinal strain (ΔGLS, percentage points) from baseline to 12 months in younger (<45 years, *n* = 30) and older (≥45 years, *n* = 60) cohorts. Horizontal orange lines represent median values, green triangles indicate mean values, boxes denote the interquartile range (IQR), and whiskers denote the range limits. Circular markers reflect individual patient measurements. The between-group difference was assessed using the Mann–Whitney U test (*p* = 0.002). ΔGLS, change in GLS from baseline.

Absolute GLS values at 12 months were not significantly different between younger and older patients (−16.3 [−18.4 to −13.9]% vs. −15.3 [−17.0 to −13.4]%, *p* = 0.555) (Figure 3). When expressed as a relative percentage of the baseline value, younger patients achieved a significantly greater relative strain enhancement at 12 months compared to the older group (36.3% vs. 19.3%, *p* < 0.001).

The earlier differences in TAPSE and mean E/E′ ratio were attenuated by 12 months. TAPSE was 22 [20–22.8] mm in younger patients and 21 [19–22] mm in older patients (*p* = 0.358), while the mean E/E′ ratio remained low and comparable between groups (6.9 [6.2–7.8] vs. 7.0 [6.0–8.4], *p* = 0.543). LVOT VTI showed no significant age-related difference at 12 months (18.7 [17.0–20.7] cm vs. 17.8 [16.2–20.6] cm, *p* = 0.148) (Table 3).

Although absolute GLS values at 12 months were similar between younger and older patients (*p* = 0.555), the magnitude of GLS improvement from baseline to 12 months (ΔGLS) differed significantly between groups. Younger patients showed greater longitudinal strain recovery than older patients (ΔGLS −3.7 [−5.1 to −1.9] vs. −2.3 [−3.0 to −1.3] percentage points, *p* = 0.002, Mann–Whitney U test). This disparity was characterized by a moderate-to-large true effect size (Mann–Whitney), confirming that the variation in deformation pathways is robust. A similar divergence was observed earlier, with younger patients achieving greater ΔGLS improvement from baseline to 6 months (−3.1 [−4.1 to −1.4] vs. −1.2 [−1.8 to −0.7] percentage points, *p* < 0.001). Although absolute GLS values did not differ significantly between groups at baseline or at 12 months, younger patients demonstrated a significantly greater within-patient improvement in GLS over time. These findings indicate different longitudinal recovery trajectories despite convergence toward similar cross-sectional GLS values at follow-up.

### 3.3. Exploratory Association Between Prescribed Medication Regimens and LVEF Trajectory

In the overall cohort, HF-GDMT (guideline-directed medical therapy for heart failure) intensity was positively associated with 12-month LVEF recovery after AMI. The HF-GDMT score correlated significantly with the absolute change in LVEF from baseline to 12 months (Spearman ρ = 0.36, *p* < 0.001; *n* = 90).

In age-stratified exploratory analyses, this association was nominally present only in the older cohort (ρ = 0.59, *p* < 0.001; *n* = 60), with no correlation observed in the younger group (ρ = −0.05, *p* = 0.788; *n* = 30). However, this apparent correlation is strictly descriptive and intrinsically confounded by baseline functional severity: older patients prescribed 3–4 GDMT classes presented with a significantly lower baseline LVEF compared to those receiving 0–2 classes (37.4% vs. 49.3%, *p* < 0.001), thus conferring a substantially larger mathematical margin for absolute ejection fraction recovery rather than demonstrating a verified pharmacological dose–response effect.

**Figure 4:**
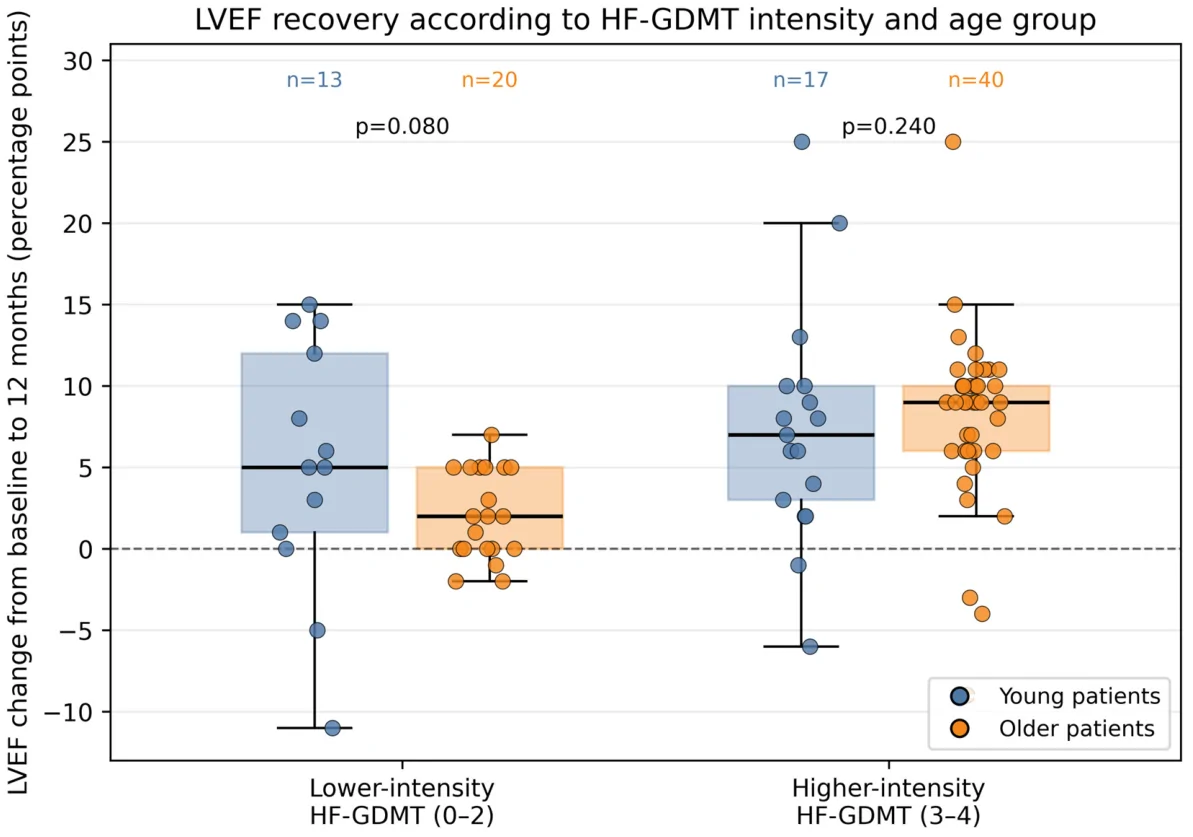
Age-stratified association between heart failure guideline-directed medical therapy intensity and 12-month left ventricular ejection fraction recovery. Box plots display 12-month LVEF according to lower-intensity (0–2 drug classes) versus higher-intensity (3–4 drug classes) HF-GDMT in younger (blue dots) and older (orange dots) patients. The horizontal solid line within boxes marks the median, box limits represent the 25th to 75th percentiles, and whiskers depict total data dispersion. The dashed reference line at 0 indicates no change from baseline LVEF. Between-group subgroup comparisons were performed using the Mann–Whitney U test. LVEF, left ventricular ejection fraction; HF-GDMT, guideline-directed medical therapy for heart failure.

When HF-GDMT intensity was grouped as lower intensity, defined as 0–2 drug classes, versus higher intensity, defined as 3–4 drug classes, patients receiving higher-intensity therapy showed greater LVEF improvement. In the lower-intensity group, median LVEF recovery was 5.0 [1.0–12.0] percentage points in younger patients and 2.0 [0.0–5.0] percentage points in older patients (*p* = 0.080). In the higher-intensity group, median LVEF recovery was 7.0 [3.0–10.0] percentage points in younger patients and 9.0 [6.0–10.0] percentage points in older patients (*p* = 0.240) (Figure 4).

Baseline characteristics according to GDMT intensity are presented in Table 4. Among patients aged ≥45 years, those receiving higher-intensity GDMT (3–4 drug classes) had significantly lower baseline LVEF compared with those treated with 0–2 GDMT classes (37.4% vs. 49.3%, *p* < 0.001). In contrast, baseline LVEF did not differ significantly between GDMT-intensity groups in younger patients (40.8% vs. 45.6%, *p* = 0.121). Baseline NT-proBNP, serum creatinine, and hs-cTnI were comparable across GDMT-intensity strata in both age groups.

Although patients attended scheduled follow-up visits with documented continuation of their discharge prescriptions, the observed association between higher HF-GDMT class counts and nominal LVEF change must be interpreted strictly as exploratory, reflecting baseline functional disparities (Table 4) rather than a verified dose-dependent treatment effect. The present study was designed to evaluate longitudinal myocardial functional recovery after AMI using conventional and deformation imaging rather than clinical outcomes. Therefore, the prognostic implications of the observed age-related differences remain uncertain and should be interpreted as hypothesis-generating until confirmed by dedicated outcome studies.

## 4. Discussion

In this prospective cohort study of patients with acute myocardial infarction undergoing primary percutaneous coronary intervention, we have observed several key findings. Older patients had longer pain-to-balloon times despite similar cardiovascular risk factors and STEMI presentation rates compared with younger patients. The longer the delay, the higher the admission levels of hs-cTnI and NT-proBNP, indicating a worse baseline biomarker profile associated with cardiomyocyte injury and myocardial wall stress, but not definitive for total infarct size, especially in the absence of serial peak biomarker kinetics or CMR imaging. Over 12 months recovery of left ventricular systolic function was similar in the age groups, but differences were observed in right ventricular longitudinal function and left ventricular filling pressures in the early phase after infarction. Furthermore, older patients prescribed higher numbers of HF-GDMT drug classes showed greater nominal LVEF increases, an exploratory pattern largely reflecting their significantly lower baseline LVEF rather than a demonstrated causal treatment effect.

The present study showed that older patients had longer pain-to-balloon times, largely due to pre-hospital delays especially the symptom-to-door time. Conversely, door-to-balloon times at our dedicated cardiovascular center were consistently <60 min for both age groups. Of note, the total ischemic time was 720 min for the older patients and 540 min for the younger patients (*p* = 0.021), indicating delays in care-seeking behaviors rather than hospital inefficiencies. Forty-five percent of the older patients presented after the ideal 12-h intervention window compared with 20% of the younger patients. All patients received emergency treatment, but older patients presented later and had worse biomarker profiles and heart function. Thus, the duration of coronary occlusion and patient behavior were more important factors than any biological differences related to age. Elevated hs-cTnI and NT-proBNP levels in the older comparator group reflect a more adverse acute presentation. Although elevated NT-proBNP indicates hemodynamic and neurohormonal wall stress during acute ischemia, this large difference (4776 vs. 834 pg/mL) should not be interpreted as reflecting infarct-related myocardial stress alone. In an age-stratified observational cohort, baseline natriuretic peptide concentrations are heavily influenced by physiological aging, subclinical alterations in left ventricular compliance, subtle baseline diastolic properties, and age-dependent renal clearance kinetics, rather than acute ischemic insult in isolation [9,14,15]. However, the study limitations, including the lack of peak hs-cTnI, cumulative troponin release, CK/CK-MB kinetics, or CMR imaging data, preclude the use of these biomarker differences to definitively assess infarct size or to conclude that older patients have larger infarcts. This underscores the necessity of public health campaigns aiming to reduce pre-hospital delays in older adults without assuming a continued age-related decline in myocardial healing capacity.

Previous studies have shown that prolonged pain-to-balloon time is associated with impaired left ventricular functional recovery and worse clinical outcomes after STEMI. A prolonged pre-balloon time (PPBT) correlates with diminished left ventricular ejection fraction, elevated in-hospital complications, and heightened 30-day mortality. Multivariate Cox regression demonstrates that each additional hour of PPBT, together with PPBT over 360 min (hazard ratio 1.6), independently elevates the one-year death risk. A retrospective analysis revealed no significant differences in mortality or major adverse cardiovascular and cerebrovascular events (MACCE) between STEMI and NSTEMI patients at 36 months, although the prolonged PBT group was older. Enhanced outcomes in STEMI patients were associated with reduced door-to-balloon durations when prehospital delays were below 120 min, while extended delays diminished the significance of timeliness. The duration of myocardial ischemia is directly related to the time required to arrive at the hospital [4,16,17]. A recent study that retrospectively analyzed consecutive patients aged ≤45 years who underwent coronary angiography for acute coronary syndromes revealed that in-hospital mortality was 9.9% and was associated with cardiogenic shock, severely reduced left ventricular ejection fraction, no-reflow/slow-flow phenomena, and prolonged door-to-balloon time [18].

The findings suggest small early subclinical differences in right ventricular longitudinal indices rather than clinically overt right ventricular dysfunction. Initial assessments revealed a lower right ventricular longitudinal function in the older cohort, alongside elevated left ventricular filling pressures indicated by a higher mean E/E′ ratio. The prevalence of RCA myocardial infarction was balanced between the young and older groups, ruling out an anatomical imbalance as the primary driver for delayed RV recovery. The right ventricular performance was stable in both young and old groups during the 12-month follow-up. Cross-sectional analyses showed a marginal decrease in systolic measurements for the older group at baseline (20 vs. 21.5 mm, *p* = 0.034) and 6 months (20.5 mm vs. 22 mm, *p* = 0.012). However, longitudinal changes were small (median change 0.5 mm younger; 1 mm older) and within clinically insignificant ranges. Statistical analyses showed no significant changes in the younger group and any nominal changes in the older group did not exceed the pre-established variability thresholds. Right ventricular longitudinal function was preserved after primary PCI in both demographics suggesting no significant biological remodeling or recovery differences.

Serial TAPSE measurements were within normal limits in both groups studied, with minor changes during follow-up, without a clear age-related pattern of right ventricular functional recovery. This pattern underscores the need for ongoing surveillance of right ventricular function in the older comparator group following an acute myocardial infarction. The reviewed literature on the prognostic implications of TAPSE was performed in a cohort with a median age of 59.0 years and follow-up for more than 8 years, without specific outcomes for younger age groups or detailed TAPSE dynamics. Baseline TAPSE was an independent predictor of MACE and mortality in patients with inferior ST-segment myocardial infarctions. Furthermore, decreased TAPSE was associated with a higher risk of 30-day all-cause mortality and cardiogenic shock in RCA-STEMI patients [8,19,20].

The E/E′ ratio indicated that the myocardial reserve was better in the younger patients than in the older patients. However, the diastolic function improved in both groups especially in the first 6 months after AMI. E/E′ values were similar at about 7.0 after one year, indicating effective recovery in the older patients despite initial challenges in myocardial relaxation. E/E′ is a strong predictor of outcomes in patients aged ≥45 years with STEMI and preserved LVEF after primary PCI and is associated with increased mortality and cardiovascular events. An elevated mitral annular E/E′ ratio of ≥15 within 24 h of admission was associated with in-hospital mortality in acute STEMI and it was a significant predictor for MACE [21,22]. Baseline TAPSE and E/E′ differed statistically between groups; however, the absolute values remained within normal or low-risk ranges. Therefore, these findings should not be interpreted as clinically overt right ventricular impairment or elevated filling pressures, but rather as small subclinical differences in echocardiographic indices.

A notable finding of the research is the recovery dynamics of LVEF over 12 months, with similar acute systolic dysfunction in younger and older patients and a median LVEF of 45% at enrollment. At 6 months, median LVEF was 50% for both groups (*p* = 0.473) and remained stable at 12 months. However, individual variations in persistent negative remodeling were observed in some patients. LV remodeling with LVEF < 50% at six months post-acute myocardial infarction was the only predictor of all-cause mortality. Furthermore, patients with improvement in LVEF at 1 year after AMI had better long-term outcomes, as indicated by lower rates of MACE and adverse LV remodeling versus patients with no change. During a 2-year follow-up, patients with acute myocardial infarction and mildly reduced EF exhibited superior prognoses compared to those with reduced EF, although they showed no significant differences from individuals with preserved EF [5,23,24].

Our exploratory sub-analysis underscores the need for standardized definitions when interpreting structural changes in HF. We focused on patients with real initial systolic degradation (LVEF ≤ 40%, *n* = 31) instead of general HF recovery terms for the entire cohort. Of note, the transition rate to a validated HFimpEF phenotype was high among younger individuals (25–44 years) after AMI (66.7%) but greater than two-thirds of older patients (≥45 years; 68.2%) persisted with HFrEF after 12 months despite similar medical management. It indicates that the recovery potential of older adults may be lower than that of younger patients. This divergence could be explained by reduced microvascular resilience or pre-existing structural changes in middle-aged individuals, but the precise cellular drivers remain unknown due to the absence of tissue characterization. Additional validation with advanced imaging of myocardial fibrosis is needed.

Ventricular remodeling after myocardial infarction is a major determinant of the risk of HF and its prognosis. Therefore, prevention or reversal of ventricular remodeling is essential to reduce the mortality in patients with HF. While established guidelines emphasize comprehensive GDMT after revascularization [12,25] the association observed in our cohort between prescribed drug-class count and 12-month LVEF change reflects clinical prescription patterns and baseline disease severity rather than causality. Older patients who received more aggressive therapy showed larger absolute increases in LVEF, and those with lower baseline LVEF had greater scope for improvement. This pattern suggests that the observed association may partly reflect confounding by indication and greater room for improvement among patients with lower baseline LVEF, rather than a direct causal effect of treatment intensity.

Conversely, the absence of a significant association in younger patients may reflect a less heterogeneous baseline dysfunction pattern and reinforces the need to interpret treatment-intensity analyses as exploratory rather than causal. These findings represent an exploratory association between prescribed treatment intensity and LVEF change rather than a causal treatment effect. Because patients receiving higher-intensity therapy (3–4 classes) had substantially lower baseline LVEF (37.4% vs. 49.3%), they had greater potential for absolute EF improvement. Consequently, this relationship is strongly susceptible to confounding by indication and regression to the mean/baseline functional deficit, and counting prescribed drug classes does not directly measure true adherence or target-dose titration [26]. A global retrospective study on patients with HFrEF categorized them into four groups based on the number of classes of GDMT administered. Only 20% received quadruple therapy, with each additional heart failure treatment significantly associated with decreased mortality [27]. Moreover, another retrospective study analyzing 527 patients with new-onset heart failure after AMI and PCI, showed that GDMT significantly reduced all-cause mortality and hospitalization at 12 months, as well as cardiac adverse events and readmission rates. This study emphasizes the significance of GDMT in preventing adverse ventricular remodeling and improving patient outcomes after AMI [28].

In our cohort, a substantial proportion of older patients with reduced baseline LVEF were prescribed multi-class regimens, aligning with contemporary recommendations to implement comprehensive secondary prevention regardless of age [12,25,29,30,31]. However, because our dataset tracked the count of drug classes rather than longitudinal dose titration, tolerability, or biomarker-guided titration, these patterns reflect baseline prescription strategy rather than optimized titration pathways and should be interpreted strictly as exploratory baseline prescription practices.

Serial GLS assessment provides additional information on myocardial dysfunction beyond the traditional LVEF, especially in younger patients. Both younger and older patients in the studied cohort had low GLS at baseline, but the median baseline GLS was less negative in the younger patients, suggesting potentially more ischemia at baseline. The absolute GLS values at 6 and 12 months were similar between age groups, but younger patients had a significant increase in ΔGLS with a relative percentage increase of 36.3% compared with 19.3% in older patients. This suggests better myocardial and microvascular reserve in younger patients, as opposed to reduced longitudinal reversibility in older patients post-AMI. At 6 months (*p* < 0.001) and 12 months (*p* = 0.002) statistically significant differences in longitudinal strain recovery were observed. Although the two age groups had similar static GLS endpoints within 12 months, the underlying healing kinetics differed. Although younger patients demonstrated a significantly greater relative and absolute improvement in GLS over 12 months, this should be interpreted cautiously as a signal of differing recovery trajectories rather than definitive proof of superior long-term intrinsic myocardial repair, particularly in the absence of direct infarct size quantification (e.g., CMR or peak biomarker kinetics). Younger patients had earlier improvement in longitudinal strain, consistent with benefits in early microvascular recruitment and resolution of subendocardial stunning, rather than persistent differences in mechanical function. Therefore, differences in GLS improvement are reflective of different recovery trajectories and not proof of persistent differences in absolute myocardial function. The mechanisms underlying these findings remain speculative in the absence of tissue characterization.

Previous evidence has shown that both baseline and follow-up measures of GLS serve as independent long-term predictors of adverse cardiovascular events after myocardial infarction, emphasizing its role as a sensitive marker of myocardial deformation pathways. Specifically, stable or improving GLS at one year has been associated with substantially higher 10-year survival, whereas a relative decline exceeding 7% identifies a high-risk subgroup. However, as recently demonstrated in the REDUCE-AMI sub-study by Mars et al. [31], the independent prognostic threshold of baseline GLS may become attenuated in specific subgroups presenting with preserved ejection fractions, highlighting that longitudinal deformation parameters should be interpreted with caution depending on the baseline systolic case mix [6,20,32,33,34,35].

Overall, the study supports a multimodal approach to post-AMI assessment, highlighting the effect of presentation timing and baseline differences. Older patients did not differ in frequency of STEMI or 12-month recovery metrics compared to younger ones, but had longer pre-hospital ischemic delays associated with higher levels of biomarkers and altered biventricular performance. Thus, operational factors and early functional decline influence recovery more than age-related biological changes. The study suggests pre-hospital ischemic time, cardiac biomarkers, echocardiography and secondary prevention to be included in the risk assessment and treatment after primary PCI. This study has several limitations. First, our results are inherently limited by the modest sample size (*n* = 90) and single-center design in terms of statistical power and external generalizability. The small sample size, especially in the young AMI group (*n* = 30), did not allow for robust multivariable regression modeling to fully account for potential confounding factors at baseline (e.g., sex distribution, specific comorbidity profiles, exact ischemic time intervals) and increased the risk of Type II statistical errors for minor differences between groups. Finally, all subjects were recruited from a single tertiary high-volume cardiovascular center with standardized door-to-balloon times <60 min; therefore, the results and the trajectory patterns observed may not be fully representative of more heterogeneous community hospital settings or multi-center cohorts with different reperfusion systems. Thus, all age-stratified observations and functional comparisons should be interpreted only as exploratory and hypothesis-generating, providing a baseline framework to be confirmed in large-scale, prospective multicenter cohorts. Second, the current analysis was limited to functional remodeling, as assessed by serial echocardiography. Adjusted clinical outcome end points were beyond the scope of the current analysis, although clinical follow-up data were collected and will be reported separately. Third, infarct size was not directly quantified because the protocol did not systematically collect peak biomarker kinetics or CMR imaging data; thus, admission biomarker elevations should not be interpreted as direct evidence of a larger infarct mass. Finally, GDMT intensity was pragmatically measured as the number of prescribed prognostic drug classes without assessment of target-dose titration, tolerability, or objective pharmacy-refill adherence. Combined with the substantial baseline LVEF imbalance across GDMT groups (Table 4), these treatment-related analyses are strictly descriptive and hypothesis-generating, subject to confounding by indication and mathematical room for recovery. Future research should validate these findings in larger multicenter cohorts with longer follow-up and adjudicated clinical endpoints. Further studies should evaluate GDMT initiation, titration, target-dose achievement, and long-term clinical endpoints in larger multicenter cohorts to clarify whether treatment-intensity optimization has differential benefits according to age and baseline ventricular phenotype after AMI.

## 5. Conclusions

In this exploratory cohort, 12-month conventional systolic recovery assessed by LVEF and absolute GLS was comparable between younger and older patients undergoing primary PCI. Although older patients presented with longer pre-hospital ischemic delays and elevated admission biomarkers reflecting acute cardiomyocyte injury and wall stress, these observations cannot be interpreted as definitive evidence of larger infarct size due to the absence of peak biomarker kinetics or CMR imaging data. Younger patients exhibited a greater longitudinal GLS improvement trajectory over time; however, given the lack of direct infarct size quantification and tissue characterization, all conclusions regarding age-related myocardial recovery must remain cautious and hypothesis-generating until validated by studies incorporating comprehensive structural and tissue imaging. Additionally, associations between medication class counts and LVEF change should be interpreted with caution given the marked baseline systolic imbalances across treatment strata.

## Figures and Tables

**Table 1:** Patient characteristics at baseline—pain to balloon time (in minutes).

Variable	Young Patients (*n* = 30 Patients)	Older Patients (*n* = 60 Patients)	*p*-Value
Pain-to-balloon time < 360 min	1 (3.3%)	7 (11.7%)	0.260
Pain-to-balloon time—360–720 min	23 (76.7%)	26 (43.3%)	0.003
Pain-to-balloon time > 720 min	6 (20%)	27 (45%)	0.022

**Table 2:** Patient characteristics at baseline.

Variable	Young Patients (*n* = 30 Patients)	Older Patients (*n* = 60 Patients)	*p*-Value
Demographic Characteristics and Cardiovascular Risk Factors			
Age, years	42 [40–43]	59 [52.8–67.2]	<0.001
Female sex, *n* (%)	4 (13.3)	17 (28.3)	0.113
Hypertension, *n* (%)	10 (33.3)	26 (43.3)	0.361
Obesity, *n* (%)	7 (23.3)	17 (28.3)	0.613
Body mass index, kg/m^2^	26.9 [25.1–28.2]	27.5 [25.4–30.1]	0.423
Known dyslipidemia, *n* (%)	3 (10.0)	14 (23.3)	0.128
Diabetes mellitus, *n* (%)	4 (13.3)	8 (13.3)	1.000
Smoking, *n* (%)	23 (76.7)	47 (78.3)	0.858
Alcohol consumption, *n* (%)	10 (33.3)	14 (23.3)	0.312
Drug use, *n* (%)	1 (3.3)	0 (0.0)	0.333
STEMI presentation, *n* (%)	25 (83.3)	50 (83.3)	1.000
Heart rate (bpm), mean ± SD	98.96 ± 11.56	73.87 ± 11.75	<0.001
SBP (mmHg), mean ± SD	132.60 ± 15.65	146.28 ± 14.58	<0.001
DBP (mmHg), mean ± SD	85.52 ± 10.58	82.19 ± 10.15	0.159
Killip class ≥ II, *n* (%)	7 (23.3)	17 (28.3)	0.613
Pain-to-balloon time, min	540 [420–660]	720 [480–1230]	0.021
hs-cTnI, pg/mL	914.5 [171.5–4213.5]	2757.5 [2044.0–4045.3]	0.045
NT-proBNP, pg/mL	833.5 [95.7–1939.5]	4776.0 [3013.3–5952.0]	<0.001

DBP, diastolic blood pressure; hs-cTnI, high sensitivity cardiac troponin I; NT-proBNP, N-terminal pro B-type natriuretic peptide; SBP, systolic blood pressure; STEMI, ST-segment elevation myocardial infarction; min, minutes.

**Table 3:** Echocardiographic characteristics at baseline, 6 months and 12 months.

Variable	Young Patients (*n* = 30 Patients)	Older Patients (*n* = 60 Patients)	*p*-Value
**Baseline**			
LVEF, %	45 [40–50]	45 [38–50]	0.500
GLS, %	−11.8 [−13.1 to −10.5]	−11.2 [−13.2 to −9.2]	0.144
TAPSE, mm	21.5 [19–23]	20 [17–22]	0.034
Mean E/E′ ratio	7.5 [6.5–10.1]	8.4 [7.3–11.2]	0.042
LVOT VTI, cm	20.0 [17.3–22.4]	18.6 [16.1–21.1]	0.063
**6 Months**			
LVEF, %	50 [45–55]	50 [42–55]	0.473
GLS, %	−16.0 [−17.6 to −13.8]	−14.5 [−16.4 to −12.5]	0.114
TAPSE, mm	22 [20.2–23]	20.5 [18–22.2]	0.012
Mean E/E′ ratio	6.9 [6.4–8.1]	7.2 [5.9–8.7]	0.969
LVOT VTI, cm	19.4 [17.6–21.9]	18.1 [15.7–21.4]	0.264
**12 Months**			
LVEF, %	50 [45–55]	50 [45–55]	0.777
GLS, %	−16.3 [−18.4 to −13.9]	−15.3 [−17.0 to −13.4]	0.555
TAPSE, mm	22 [20–22.8]	21 [19–22]	0.358
Mean E/E′ ratio	6.9 [6.2–7.8]	7.0 [6.0–8.4]	0.543
LVOT VTI, cm	18.7 [17.0–20.7]	17.8 [16.2–20.6]	0.148
**Change from baseline**			
ΔGLS baseline → 6 months, %	−3.1 [−4.1 to −1.4]	−1.2 [−1.8 to −0.7]	<0.001
ΔGLS baseline → 12 months, %	−3.7 [−5.1 to −1.9]	−2.3 [−3.0 to −1.3]	0.002
Relative GLS improvement, %	36.3 [17.0–82.4]	19.3 [10.0–30.6]	<0.001

GLS, global longitudinal strain; LVEF, left ventricular ejection fraction; TAPSE, tricuspid annular plane systolic excursion; E/E′, ratio of early mitral inflow velocity to early diastolic mitral annular velocity; LVOT VTI, left ventricular outflow tract velocity–time integral; ΔGLS, change in GLS from baseline. Values are expressed as median [IQR]. *p*-values from Mann–Whitney U test.

**Table 4:** Baseline and 12-Month Left Ventricular Ejection Fraction According to Guideline-Directed Medical Therapy Intensity in the Overall Cohort (*n* = 90).

Parameter	Young Patients GDMT 0–2 (*n* = 13 Patients)	Young Patients GDMT 3–4 (*n* = 17 Patients)	*p*-Value	Older Patients GDMT 0–2 (*n* = 20 Patients)	Older Patients GDMT 3–4 (*n* = 40 Patients)	*p*-Value
**Baseline LVEF (%)**	45.6	**40.8**	**0.121**	**49.3**	**37.4**	**<0.001**
**12-month LVEF (%)**	**52.7**	**46.0**	**0.002**	**53.1**	**45.4**	**<0.001**

## Data Availability

De-identified study data are available on reasonable request from the corresponding author (larisa.anghel@umfiasi.ro). A justification for its further use should be provided.

## Abbreviations

The following abbreviations are used in this manuscript:

AMI	Acute myocardial infarction
BMI	Body mass index
CMR	Cardiac magnetic resonance
D2B	Door-to-balloon
E/E′	Ratio of early mitral inflow velocity to early diastolic mitral annular velocity
GDMT	Guideline-directed medical therapy
GLS	Global longitudinal strain
HF	Heart Failure
HF-GDMT	Guideline-directed medical therapy for heart failure
HFimpEF	Heart failure with improved ejection fraction
HFrEF	Heart failure with reduced ejection fraction
hs-cTnI	High-sensitivity cardiac troponin I
LDL-C	Low-density lipoprotein cholesterol
LVEF	Left Ventricular Ejection Fraction
LVOT VTI	Left Ventricular Outflow Tract Velocity–Time Integral
MACCE	Major adverse cardiovascular and cerebrovascular events
MACE	Major adverse cardiovascular events
NSTEMI	Non-ST-elevation myocardial infarction
NT-proBNP	N-terminal pro B-type natriuretic peptide
PCI	Percutaneous coronary intervention
PPBT	Prolonged pre-balloon time
STEMI	ST-elevation myocardial infarction
TAPSE	Tricuspid annular plane systolic excursion
ΔGLS	Change in GLS from baseline
ΔLVEF	Change in LVEF from baseline
